# The Role of Phosphatidylethanolamine N-Methyltransferase (*PEMT*) and Its Waist-Hip-Ratio-Associated Locus rs4646404 in Obesity-Related Metabolic Traits and Liver Disease

**DOI:** 10.3390/ijms242316850

**Published:** 2023-11-28

**Authors:** Chang Sun, David J. F. Holstein, Natalia Garcia-Cubero, Yusef Moulla, Christine Stroh, Arne Dietrich, Michael R. Schön, Daniel Gärtner, Tobias Lohmann, Miriam Dressler, Michael Stumvoll, Matthias Blüher, Peter Kovacs, Esther Guiu-Jurado

**Affiliations:** 1 Medical Department III—Endocrinology, Nephrology, Rheumatology, University of Leipzig Medical Center, 04103 Leipzig, Germany; 2 Clinic for Visceral, Transplantation and Thorax and Vascular Surgery, University Hospital Leipzig, 04103 Leipzig, Germany; 3 Department of General, Abdominal and Pediatric Surgery, Municipal Hospital, 07548 Gera, Germany; 4 Städtisches Klinikum Karlsruhe, Clinic of Visceral Surgery, 76133 Karlsruhe, Germany; 5 Municipal Clinic Dresden-Neustadt, 01129 Dresden, Germany; 6 Helmholtz Institute for Metabolic, Obesity and Vascular Research (HI-MAG) of the Helmholtz Zentrum München at the University of Leipzig and University Hospital Leipzig, 04103 Leipzig, Germany; 7 Deutsches Zentrum für Diabetesforschung e.V., 85764 Neuherberg, Germany

**Keywords:** *PEMT*, rs4646404, adipose tissue, fat distribution, obesity, liver disease

## Abstract

In previous genome-wide association studies (GWAS), genetic loci associated with obesity and impaired fat distribution (FD) have been identified. In the present study, we elucidated the role of the *PEMT* gene, including the waist–hip-ratio-associated single nucleotide polymorphism rs4646404, and its influence on obesity-related metabolic traits. DNA from 2926 metabolically well-characterized subjects was used for genotyping. *PEMT* expression was analyzed in paired visceral (vis) and subcutaneous (sc) adipose tissue (AT) from a subset of 574 individuals. Additionally, *PEMT* expression was examined in vis, sc AT and liver tissue in a separate cohort of 64 patients with morbid obesity and liver disease. An in vitro *Pemt* knockdown was conducted in murine epididymal and inguinal adipocytes. Our findings highlight tissue-specific variations in *PEMT* mRNA expression across the three studied tissues. Specifically, vis *PEMT* mRNA levels correlated significantly with T2D and were implicated in the progression of non-alcoholic steatohepatitis (NASH), in contrast to liver tissue, where no significant associations were found. Moreover, sc *PEMT* expression showed significant correlations with several anthropometric- and metabolic-related parameters. The rs4646404 was associated with vis AT *PEMT* expression and also with diabetes-related traits. Our in vitro experiments supported the influence of *PEMT* on adipogenesis, emphasizing its role in AT biology. In summary, our data suggest that *PEMT* plays a role in regulating FD and has implications in metabolic diseases.

## 1. Introduction

For decades, the prevalence of obesity and its medical and socioeconomic sequalae have been steadily increasing [1]. Obesity is strongly associated with the development of metabolic syndrome, including its typical cardiovascular comorbidities, accompanied by a reduced life expectancy. To date, the underlying mechanisms that predispose certain individuals to obesity and its associated comorbidities remain elusive. However, it is clear that the distribution of fat between visceral (vis) and subcutaneous (sc) adipose tissue (AT) is more decisive for the development of diabetes mellitus and cardiovascular disease than the absolute fat mass itself [2]. Many studies implicate genetics as playing a key role regarding the development of obesity and impaired fat distribution (FD) [3,4,5,6].

Using genome-wide association studies (GWAS), a large number of potential candidate genes related to adiposity, adipogenesis and especially to impaired FD have been discovered. Shungin et al. described, among others, an association with altered waist–hip ratio (WHR), even after adjustment for body mass index (BMI), for the gene *Phosphatidylethanolamine N-methyltransferase* (*PEMT*) and its single nucleotide polymorphism (SNP), rs4646404 [7]. *PEMT*, which is resident on chromosome 17, is expressed in AT and liver tissue, where it catalyzes three sequential steps in phosphatidylcholine (PC) and phospholipid biosynthesis [8]. *PEMT*-mediated alterations in the PC/ phosphatidylethanolamine (PE) ratio are associated with the development and progression of non-alcoholic fatty liver disease (NAFLD) and its successor, non-alcoholic steatohepatitis (NASH), as well as liver failure and impaired liver regeneration in humans [9,10]. This might be due to increased triacylglyceride (TAG) storage as a result of impaired very-low-density lipoproteins (VLDL) secretion. Moreover, a reduced PC/PE ratio leads to impaired membrane integrity with regard to membrane permeability and cellular damage [9]. Furthermore, it has been shown that lower hepatic *PEMT* expression is correlated with the severity of liver damage in patients with NAFLD. However, none of the SNPs analyzed were associated with hepatic *PEMT* expression [11]. Moreover, *PEMT*-deficient mice gained little, or no weight while being fed a high-fat diet (HFD) yet developed fatty liver disease and showed defective VLDL secretion. This can be reversed through dietary choline supplementation, further underlining the central role of *PEMT* in mediating metabolism and cellular homeostasis [12]. Studies of hepatic *Pemt* activity versus extrahepatic expression in relation to diet-induced obesity (DIO) and insulin resistance (IR) have shown that hepatic *Pemt* activity seems to be the determining factor for the development of DIO and IR, as *Pemt* deficient mice are protected against DIO and IR. However, these mice developed NASH and showed a reduced PC/PE ratio, which in turn explains an increased insulin sensitivity in hepatocytes [13]. Transcriptional upregulation of *PEMT* in human AT showed a correlation with WHR adjusted for BMI, indicating its association with an increased genetic risk for obesity [14]. Studies focusing on phospholipid metabolism in mice demonstrated the regulatory role of *Pemt* during the induction and differentiation of early and late adipogenesis, pointing to its possible role in the development of adiposity [15]. 

Taking into consideration the potential impact of *PEMT* on the aforementioned biological processes, the expression of *PEMT* was assessed in human vis and sc AT, as well as in liver tissue, according to the metabolic state of participating subjects. Furthermore, the functional relevance in adipogenesis in vitro, and the analyzed causal connections between its genetic variant rs4646404 and metabolic traits, were analyzed.

## 2. Results

### 2.1. PEMT mRNA Expression in AT Correlates with Parameters of Obesity, Body Fat Distribution, Insulin Sensitivity and Circulating Adipokines

**Figure 1:**
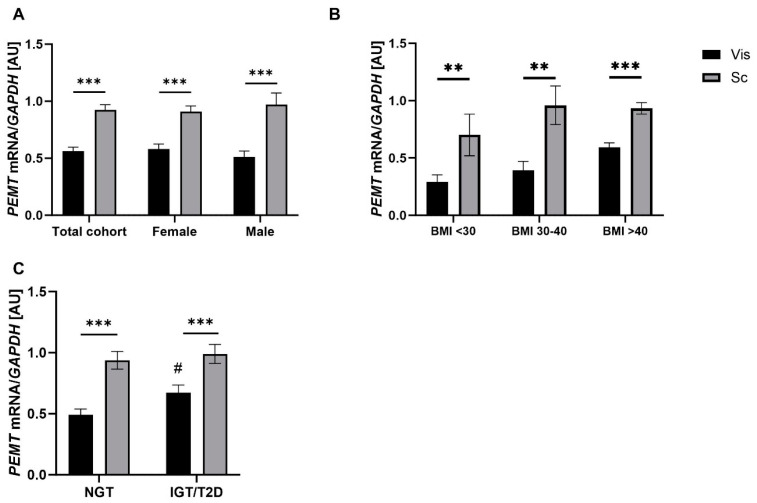
*PEMT* mRNA expression among visceral (vis) and subcutaneous (sc) adipose tissue (AT): (**A**) *PEMT* mRNA expression in 574 paired human vis and sc AT samples in the total cohort and grouped by sex (424 females, 150 males); (**B**) *PEMT* mRNA expression in paired human vis and sc AT samples grouped by obesity status (<30 kg/m^2^ N = 21, 30–40 kg/m^2^ N = 48, BMI > 40 kg/m^2^ N = 488); and (**C**) *PEMT* mRNA expression in paired human vis and sc AT samples grouped by diabetes status (NGT: subjects with normal glucose tolerance, n = 250; IGT/T2D: subjects with impaired glucose tolerance or subjects with Type 2 diabetes, n =225). Data are shown as mean ± SEM. **/*** sc vs. vis AT depot; # BMI 30–40 kg/m^2^ and BMI > 40 kg/m^2^ vs. <30 kg/m^2^ depot. # *p* < 0.05; ** *p* < 0.01; *** *p* < 0.001.

**Figure 2:**
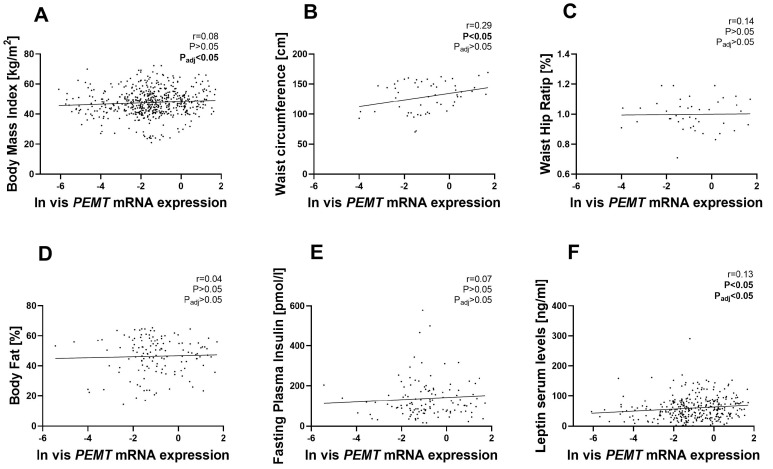
Correlation between vis *PEMT* mRNA expression and fat distribution variables: The correlation of vis *PEMT* mRNA levels with BMI ((**A**), n = 574); waist circumference ((**B**), n = 55); WHR ((**C**), n = 41); body fat% ((**D**), n = 135); fasting insulin ((**E**), n = 121); and leptin levels ((**F**), n = 337) are shown. Pearson correlation coefficients and corresponding *p*-values have been included. p_adj_ means after adjusted BMI, sex and age, except BMI traits. vis, visceral adipose tissue; BMI, body mass index.

We performed an analysis of paired vis and sc AT biopsy samples from 574 subjects. *PEMT* mRNA was found to be significantly higher in sc than in vis AT, in both females and males (Figure 1A), and vis mRNA expression correlated significantly with sc AT expression (r = 0.15, *p* < 0.001, p_adj_ = 0.001). We further stratified these paired samples into BMI categories: <30, 30–40, and >40 kg/m^2^. *PEMT* mRNA expression was significantly higher in sc AT than in vis AT in all BMI categories (Figure 1B). Notably, although the difference was not statistically significant, there was a trend towards increased *PEMT* mRNA expression in vis AT as the BMI category increased (Figure 1B). In the subsequent correlational analysis, adjusted for sex and age, we found a significant correlation between BMI and *PEMT* mRNA expression in vis AT (Figure 2A; Table S1). When comparing subjects with impaired glucose tolerance (IGT) or type 2 diabetes (T2D) to those with normal glucose tolerance (NGT), *PEMT* mRNA expression was significantly higher in vis AT of the former group (Figure 1C). Our logistic regression analyses for the T2D case–control study showed an association between IGT/T2D and vis AT *PEMT* mRNA expression (OR = 1.3, 95%CI = 1.02–1.6, *p* = 0.03). However, this association was not significant after adjusting for BMI, sex, and age. The data suggested that age, rather than obesity, was driving this association, as the association remained significant after adjusting for BMI and sex (*p* < 0.05, Table S2), but not when age was included in the statistical model. 

**Figure 3:**
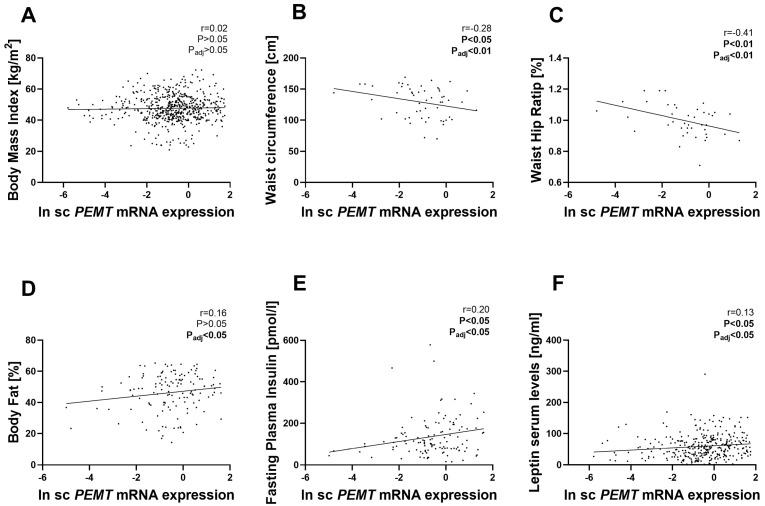
Correlation between sc *PEMT* mRNA expression and fat distribution variables: the correlation of sc *PEMT* mRNA levels with BMI ((**A**), n = 574); waist circumference ((**B**), n = 55); WHR ((**C**); n = 41), body fat% ((**D**), n = 135); fasting insulin ((**E**), n = 121); and leptin levels ((**F**), n = 337) are shown. Pearson correlation coefficients and corresponding *p*-values have been included. p_adj_ means after adjusted BMI, sex, and age, except BMI traits. sc, subcutaneous adipose tissue; BMI, body mass index.

Figure 2A illustrates the positive correlation between *PEMT* mRNA expression in vis AT and BMI, adjusted by sex and age. However, no such association was found in sc AT (Figure 3A). We also observed correlations between *PEMT* mRNA and waist circumference (WC) in vis AT (Figure 2B) and in sc AT (Figure 3B). However, after adjusting for age, sex, and BMI in multiple linear regression models, only the negative correlation in sc AT remained significant (Table S1). *PEMT* mRNA expression in sc AT showed a negative correlation with WHR, which was maintained even after adjusting for BMI, age, and sex (Figure 3C), not in vis AT (Figure 2C). Similarly, we found a positive association between *PEMT* mRNA expression in sc AT and body fat percentage, which was not observed in vis AT (Figure 2D and Figure 3D). This association in sc AT remained significant even after adjusting for sex, age, and BMI.

We further investigated the association between *PEMT* mRNA expression and various metabolic phenotypes, including parameters related to diabetes, lipids, inflammation and adipokines (Table S1). Our analysis showed that *PEMT* mRNA expression in sc AT was positively correlated with fasting insulin levels (Figure 3E) but not in vis AT (Figure 2E), and this association remained significant even in multiple linear regression models after adjusting for age, sex, and BMI (Table S1). Additionally, both vis and sc AT *PEMT* mRNA expression showed positive correlations with circulating leptin levels. These associations remained significant after adjustments for age, sex, and BMI (Figure 2F and Figure 3F).

### 2.2. Association of PEMT Expression with Metabolic Traits and Presence of NAFLD and NASH

**Figure 4:**
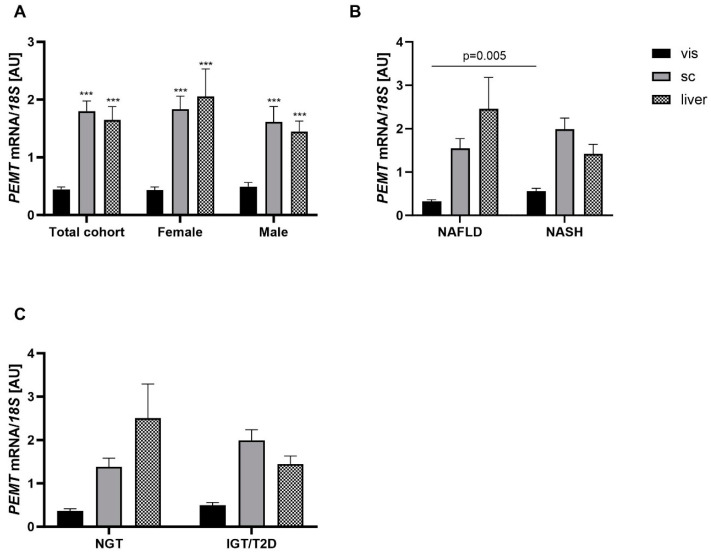
*PEMT* mRNA expression among visceral (vis), subcutaneous (sc) adipose tissue (AT) and liver tissue (LT): (**A**) *PEMT* mRNA expression in 64 paired human vis AT, sc AT and LT samples in the total cohort and grouped by sex (45 females, 19 males); (**B**) *PEMT* mRNA expression in paired human vis AT, sc AT and LT samples grouped by presence of non-alcoholic fatty liver disease (NAFLD) (n = 29) or non-alcoholic steatohepatitis (NASH) (n = 38); and (**C**) *PEMT* mRNA expression in paired human vis AT, sc AT and LT samples grouped by diabetes status (NGT: subjects with normal glucose tolerance, n = 26; IGT/T2D: subjects with impaired glucose tolerance or subjects with Type 2 diabetes, n = 38). Data are shown as mean ± SEM. *** *p* < 0.001 compared to vis AT.

We conducted *PEMT* mRNA expression analysis in paired vis AT, sc AT and liver tissue samples to study associations with metabolic phenotypes. Overall, significantly higher *PEMT* mRNA expression was observed in sc AT and liver tissue compared to vis AT among the sub-cohort studied (Figure 4A). Furthermore, our analysis indicated no sex-specific differences in *PEMT* mRNA expression in the three tissues. Contrary to the previously pronounced correlation observed in *PEMT* mRNA expression between vis and sc AT, such a correlation was absent in liver tissue (vis vs. liver, r = −0.03, *p* = 0.8; sc vs. liver, r = 0.15, *p* = 0.9). Compared to subjects without NASH, NASH patients showed significantly elevated *PEMT* mRNA expression in vis AT (Figure 4B, *p* < 0.05). In contrast, NASH patients had lower *PEMT* mRNA expression in liver tissue when compared to those with only NAFLD. Moreover, subjects with alterations in glucose homeostasis (IGT and T2D) did not show significant changes in *PEMT* mRNA expression. Albeit not significant, but consistent with our previous findings (Figure 1C), in vis AT, there was an increased expression of *PEMT* mRNA in subjects with IGT and T2D. Similarly, *PEMT* mRNA expression was also increased in sc AT, but decreased in liver tissue (Figure 4C). 

Finally, to discern the correlation between *PEMT* mRNA expression and liver disease/dysfunction across distinct tissues, we aimed to identify whether there is a single tissue important for NASH development. Our findings showed that *PEMT* mRNA expression in sc AT displayed significant associations with IGT, T2D and WC (Table 1). In contrast, vis *PEMT* mRNA expression was significantly associated with the presence of NASH (*p* = 0.01, OR = 31.28) and liver mean fat (*p* = 0.003, Table 1). Interestingly, in liver tissue, we found no association between *PEMT* mRNA expression and anthropometric or metabolic parameters. 

### 2.3. Decreased Lipid Accumulation in Pemt Knockdown in Epididymal Adipocyte Cell Line

**Figure 5:**
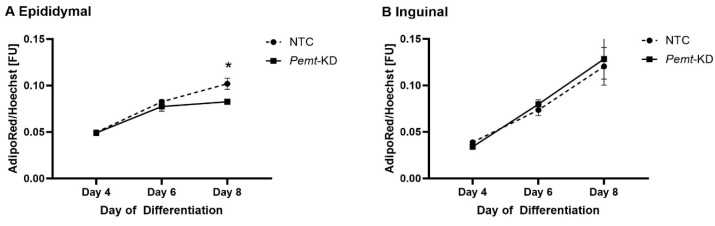
Quantification of lipid droplets by measuring the AdipoRed^TM^ fluorescence signal normalized to the Hoechst fluorescence signal. Lipid droplets quantification in: the epididymal (**A**); and the inguinal (**B**) cell lines. Lipid accumulation normalized to the Hoechst signal compared to NTC (non-silencing siRNA group). AdipoRed^TM^ stains triglycerides of the lipid droplets and Hoechst stains nuclei of cells. KD: knockdown. * *p* < 0.05.

In vitro results revealed that *Pemt* knockdown is associated with a significant decrease in lipid accumulation among epididymal AT cells when tested vs. non-silencing siRNA (NTC)-controls during adipogenesis in mice adipocytes at day 8 (*p* < 0.05). However, there was no significant alteration regarding lipid accumulation in inguinal AT cells during adipogenesis (Figure 5). 

### 2.4. Association of rs4646404 in PEMT with Fat Depot-Specific PEMT mRNA

**Figure 6:**
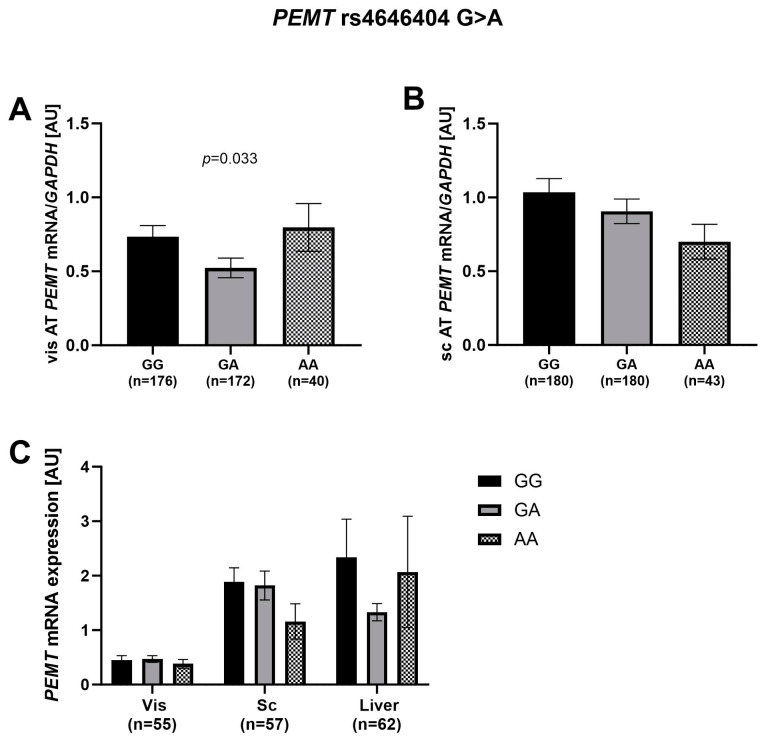
Association of rs4646404 with *PEMT* mRNA expression in (**A**) visceral (vis), (**B**) subcutaneous (sc) and (**C**) liver AT. Data are given as arithmetic mean ± SEM; *p* < 0.05 in a recessive model of inheritance. AT, adipose tissue; vis, visceral adipose tissue; sc, subcutaneous adipose tissue.

With the exception of the recessive genetic model of rs4646404, which was associated with *PEMT* mRNA levels in vis AT, no association with other genetic models of the variant with *PEMT* mRNA levels was found in either vis or sc AT (Table 2, Figure 6A). The significant *p*-value of the recessive genetic model in visceral AT did not withstand adjustment for sex, age, and BMI. Interestingly, the significant association was only in females when stratified by sex (Table 2). Although there was no statistically significant association between rs4646404 and sc *PEMT* mRNA expression, a decreasing trend with reduced *PEMT* mRNA expression in sc AT in carriers of the A allele was found (Figure 6B). Furthermore, an extended analysis was conducted with the sub-cohort to test for the association of rs4646404 with *PEMT* mRNA expression in liver tissue. We also observed a similar decreasing trend in the sub-cohort for sc AT, and the results did not indicate a significant association between SNP and *PEMT* mRNA expression in liver tissue (Figure 6C). 

### 2.5. rs4646404 and Parameters Related to Obesity, Diabetes and Fat Distribution

To clarify the relationship between rs4646404 G > A (*PEMT*) and human metabolic phenotypes, and to attempt replication of the previously described relationship of SNP on WHR, we genotyped the SNP in the entire study cohort (Table S3). The genotype distribution was in Hardy–Weinberg Equilibrium (*p* > 0.05) with a minor allele frequency reaching 32%. Although previous GWAS reported a nominally significant sexual dimorphism for rs4646404 [7], we found no sex–SNP interaction effects in the linear regression analyses in the present study. Nevertheless, we performed statistical subgroup analyses by sex, but the results remained unchanged (data not shown). 

Individuals homozygous for rs4646404 (AA) had a higher BMI, waist, body fat percentage, and fat area (vis and sc) compared to G allele carriers (Table S3). Given the association of *PEMT* mRNA with IGT/T2D, we further analyzed the relationship between rs4646404 and IGT/T2D in our cohort, which comprised 649 NGT and 534 IGT/T2D patients (Table S5). Surprisingly, we found no significant association between them (Table S1). 

However, in subgroup analyses, rs4646404 showed associations with specific metabolic parameters. For NGT patients, A allele carriers of rs4646404 had higher 120 min oGTT levels and FFA than those homozygous for the G allele (*p* < 0.05, after being adjusted for sex, age, and BMI. Table S5). On the other hand, in patients with IGT/T2D, those homozygous for the A allele showed a lower vis/sc ratio and smaller vis fat cell size than G allele carriers (*p* < 0.05, after being adjusted sex, age and BMI. Table S5). In our liver-specific cohort, no prominent associations were identified between rs4646404 and the parameters concerning diabetes and lipid metabolism (Table S4). 

## 3. Discussion

Previous GWAS have implicated the role of *PEMT* in FD. However, the complex role of *PEMT* in obesity and its related diseases remains incompletely understood. In the present study, we aimed to expand this understanding by investigating the distinct expression patterns of *PEMT* mRNA in three tissues. Moreover, we examined its association with various metabolic parameters and phenotypes. Finally, we also explored the potential influence of the *PEMT* variant, specifically SNP rs4646404, on mRNA expression in these three tissues, and its correlation with metabolic parameters and phenotypes.

The key findings are: (1) *PEMT* mRNA expression exhibits tissue-specific variations in all three tissues, being lower in vis AT than in sc and liver; (2) the *PEMT* mRNA expression in vis AT correlates with diabetes and NASH; and (3) rs4646404 is associated with vis *PEMT* mRNA expression.

### 3.1. PEMT mRNA Expression Is Fat Depot Specific and Related to Diabetes Status and NASH

It has been well established that the distribution of fat between vis and sc AT is more decisive for the development of diabetes mellitus and cardiovascular disease than the absolute fat mass itself [2]. In the present study, a significantly higher expression of *PEMT* mRNA in sc AT and liver tissue than in vis AT was observed, regardless of sex. Furthermore, the increased expression of vis *PEMT* is related to the progression of metabolic diseases (T2D and NASH). This supports the potential role of vis AT *PEMT* expression in the development of T2D. This could be explained by the fact that an excess of visceral fat is related to a greater risk of insulin resistance. Furthermore, *PEMT*, which influences lipoprotein metabolism important for lipid droplet stabilization, might be a key to this relationship, emphasizing its role in physiological FD [16,17]. Some studies have shown that during the process of (pre-) adipocyte differentiation, which involves the transformation of precursor cells into mature fat-storing cells, *PEMT* expression is strongly induced [15,18]. Consistent with these findings, our in vitro results showed a decrease in lipid accumulation following *Pemt* knockdown in epididymal adipocytes. Interestingly, a higher vis *PEMT* mRNA was associated with the presence of NASH. Although liver may be seen as the primary tissue involved in NASH development, our data suggest that vis *PEMT* mRNA expression may influence the progression from NAFLD to NASH. Elevated *PEMT* expression in vis AT may play a critical role in modulating metabolic pathways, pointing to a profound influence in metabolic syndromes. This could contribute to *PEMT*’s key role in phospholipid metabolism, catalyzing the methylation of PE to PC via the *PEMT* pathway in the liver [12]. PC is a key determinant of hepatic TAG levels and, interestingly, an imbalance of both low or high PC levels increases liver TAG by promoting distinct molecular mechanisms [19,20,21,22]. Then, by altering the PC/PE ratio, it affects membrane integrity, leading to the onset of hepatic inflammatory responses [9,23,24]. Moreover, our data highlight a significant correlation between sc *PEMT* expression and various anthropometric measures, such as WC, WHR, and body fat percentage (BF%), as well as a positive correlation with fasting insulin and leptin levels. This may not seem surprising, as it has been confirmed in an early rodent study that *Pemt* knockout rodent models are protected from diet-induced obesity and diabetes [12]. Lastly, although not reaching statistical significance in liver tissue, our data point to a lower *PEMT* mRNA expression in liver tissue in the presence of concurrent NASH and NAFLD, consistent with observations documented in a recent study [11]. Future research should deepen our understanding of the physiological and biochemical functions of ectopic fat. Although ectopic ATs (e.g., those fats stored in the liver) are associated with a higher risk of metabolic diseases and may appear similar to vis/sc ATs; however, their functional pattern is different. Not only do they have a different genetic basis for FD [6], but they also have a different expression. In our data, the expression of *PEMT* is significantly correlated between vis and sc AT, but its expression in the liver is not related to either of them. 

### 3.2. SNP rs4646404: A Potential Modulator of PEMT Expression

It has been noted that, although dietary risk factors for *PEMT* play important roles, the contribution of *PEMT* to genetic susceptibility to obesity/fat distribution is well established [25,26]. However, few loci have been linked to a molecular mechanism so far. In the present study, the WHR risk rs4646404 A-allele was nominally significantly associated with vis *PEMT* mRNA expression. For sc AT, although not significant, the A-allele carriers were found to have lower *PEMT* mRNA levels, a finding consistent with data from the Genotype-Tissue Expression (GTEx) database [27]. Furthermore, it should be noted that rs4646404 is located between two enhancer regions associated with *PEMT*, which may be involved in the regulation of *PEMT* transcription. It should be mentioned that expression quantitative trait loci (eQTL) studies may use smaller sample sizes than clinical trait association studies, as eQTLs tend to explain a greater proportion of trait variance [28]. However, we believe that adequate statistical power to detect significant associations between rs4646404 and *PEMT* mRNA expression in AT is still lacking, although the sample size of our study may be appropriate in the context of expression studies. Furthermore, we demonstrated that rs4646404 was associated with fat cell size in IGT/T2D patients (Table S5), which may support the hypothesis of previous studies in which *PEMT* polymorphism rs4646404 was associated with WHR adjusted for BMI, but not with BMI alone, indicating the gene’s role in body FD rather than overall adiposity. Lastly, *PEMT* may be mechanistically involved in the stabilization of lipid droplets and hypertrophy of adipocytes in obese human subjects, similar to that observed in established rodent models [12,14,16]. 

Several limitations of this study deserve to be acknowledged. Despite analyzing *PEMT* mRNA expression in a relatively large cohort of paired human vis and sc AT samples (N~570), we acknowledge that the sample size might still restrict the statistical power to detect weaker correlations with the phenotypes studied. Furthermore, it should be highlighted that the analyses concerning *PEMT* mRNA expression in liver and other ATs were based on a cohort of individuals with morbid obesity, all of whom were already diagnosed with NAFLD. This exclusive approach, together with a limited sample size (N = 64), may restrict the statistical power of our study, potentially masking subtler effect sizes in our results.

## 4. Materials and Methods

### 4.1. Subjects

The study cohort included 2926 metabolically well-characterized participants (Table 3) of the Leipzig Obesity BioBank, recruited through German bariatric surgery centers (Leipzig, Karlsruhe, Dresden, and Gera). All subjects underwent routine clinical phenotyping as described previously [29,30]. They had a stable weight, defined as fluctuations of <2% of body weight for at least 3 months before surgery. According to ADA criteria [31], 1204 subjects were diagnosed with IGT or T2D. Patients who had acute or chronic hepatic, inflammatory, infectious, or neoplastic diseases were excluded from the study. The study was approved by the Ethics Committee of the University of Leipzig (approval numbers: 159-12-21052012, 017-12-23012012). 

In order to study the role of *PEMT* in the development of liver disease, a sub-cohort of 64 extensively characterized subjects with sc AT, vis AT, and liver samples was used (Table 4). These patients underwent open abdominal surgery for Roux-en-Y bypass, sleeve gastrectomy, elective cholecystectomy or explorative laparotomy and their liver volume and liver fat content were assessed as described elsewhere [32]. Liver samples were obtained during surgery from the anterior margin left lobe segment 3 that was given to pathology preserved in 10% formalin. Paraffin-embedded tissue sections were stained using hematoxylin–eosin and Masson trichrome protocols. A pathologist blinded to the study analyzed the liver biopsy prospectively. The samples were scored regarding steatosis, inflammation, fibrosis, and ballooning of hepatocytes, and the NAFLD activity score according to Kleiner et al. was used [33]. Steatohepatitis was diagnosed if the patient had >5% fat in their liver; NASH was confirmed when ballooning of hepatocytes was present. The study was approved by the Ethics Committee of the University of Leipzig (Nr. 363–10-13122010) and registered at German Clinical Trial Register DRKS00000686, Universal Trial Number U1111-1119–0341). The study designs follow the Declaration of Helsinki, and all participants gave written informed consent prior to participation. 

### 4.2. PEMT mRNA Expression Analysis in AT and Liver

AT samples of abdominal omental vis and sc were obtained from 574 Caucasians (male n = 150 and female n = 424) who underwent abdominal surgery, as described previously [34]. The ages ranged from 18 to 86 years and with a BMI between 18.3 to 70 kg/m^2^. Liver samples were obtained from 64 individuals (male n = 19 and female n = 45). The ages ranged from 26 to 66 years and the BMI mean ranged from 45.93 ± 4.89 kg/m^2^. After surgery, AT and liver samples were immediately frozen in liquid nitrogen and stored at −80 °C. RNA was extracted from AT by using RNeasy Lipid Tissue Mini Kit (Qiagen, Hilden, Germany), and quantitative (q) PCR was performed as described elsewhere [34,35]. Real-time qPCR was performed with the TaqMan Assay predesigned by Applied Biosystems (Foster City, CA, USA) for the detection of *Phosphatidylethanolamine N-Methyltransferase* (*PEMT*; Hs01002999_m1), *Eukaryotic 18S rRNA* (*18S*; Hs99999901_s1) and *Glyceraldehyde 3-phosphate dehydrogenase* (*GAPDH*; Hs 02786624_g1) mRNA expression in AT. All reactions were carried out in 96-well plates using the QuantStudioTM 6 Flex System Fast Real-Time PCR system. *PEMT* mRNA expression was calculated relative to *GAPDH* mRNA expression.

### 4.3. Genotyping

Genotyping of the SNP rs4646404 was conducted according to the manufacturer’s protocol using the SNP genotyping probes (Forward: TGATTTCCTCGAGGCAGGTGCCTGGGGTAG, Reverse: CACTGGGCGGGGTCCATGAGGGCAGCTGGA, SNP site: [C/T]; Thermo Fisher Scientific, Waltham, MA, USA). To control genotyping quality, a random selection of about 5% of the samples was re-genotyped for the SNP. All genotypes matched the initial designated genotypes. Genotype distributions were in the Hardy–Weinberg equilibrium, all presenting *p* > 0.05.

### 4.4. In Vitro Pemt Knockdown in Murine Epidydymal and Inguinal Cell Lines

AT of newborn FVB mice was extracted and immortalized using the SV40 T antigen, as described in detail elsewhere [36]. These immortalized epididymal and inguinal adipocytes were cultured and differentiated according to the reported protocols [36,37]. Shortly after, cells were grown in Dulbecco’s modified Eagle’s medium (high glucose) supplemented with 20% fetal bovine serum at 37 °C and 5% CO_2_ until reaching 80% confluence. Three days prior to induction, electroporation transfection of siRNA into the cell was performed using the Neon^®^ Transfection System. Subsequently, induction was initiated by adding 0.125 mM indomethacine, 2 µg/mL dexamethasone, and 0.5 mM isobutylmethylxanthine to the growth medium for 24 h and for differentiation; the growth medium was supplemented with 20 mM insulin and 1 nM triiodthyronine. A second chemical transfection method was performed one day after the induction. In this case, the transfection Reagent DharmaFECT^®^ was used. Cells were grown for 8 days in the differentiation medium. The cells were harvested at these time points: 80% confluence (=day-2), day 0 (=day of induction), day 2, 4, 6, 8 (=2, 4, 6, 8 days after induction), and washed and frozen immediately at −80 °C until RNA or protein extraction. All differentiation lines were run in triplicate. Adipocyte differentiation and lipid droplet accumulation was monitored by AdipoRed^TM^ staining (Lonza, Basel Switzerland); siRNA for *Pemt* (Horizon, Catalog ID: L-041650-01-0005), and a negative control (ON-TARGETplus Non-Targeting Pool, Horizon, Catalog ID: D-001810-10-20) were used. *Pemt* knockdown was validated at the mRNA and protein level. The mRNA knockdown efficiency was 84% and 78% knockdown in inguinal and epididymal adipocytes, respectively.

### 4.5. Statistical Analysis

Statistical tests were performed using the IBM SPSS Statistic 29.0 software (IBM Corp., Armok, NY, USA). Normal distribution of the variables included in this study was tested prior to the statistical analyses and logarithmically transformed to achieve approximate normal distribution. Differences in mRNA expression between vis and sc AT were assessed using the paired Student’s t-test. The unpaired t-Test was used to analyze differences in AT mRNA expression among the study groups. Logistic regression analyses were performed for the association of the SNP with the obesity/diabetes status. Linear regression analyses were used to assess the relationship between genetic variants/mRNA expression levels and quantitative metabolic traits. Pearson’s correlation analyses were conducted using two-way bivariate correlations. The additive model (with genotypes coded to 0, 1, and 2) was used, and if not stated otherwise, all *p*-values are adjusted for age, sex, and BMI. Two-sided *p*-values ≤ 0.05 were considered to provide evidence for nominal association and are presented without correction for multiple testing.

## 5. Conclusions

Our findings support the role of *PEMT* in FD as well as its association with obesity and diabetes, as demonstrated by a fat depot-specific *PEMT* mRNA expression in three distinct tissues. Furthermore, *PEMT* expression shows a correlation with metabolic traits such as WHR, BF%, insulin sensitivity, and circulating adipokines. Regarding the tissue specific expression pattern of *PEMT* in humans, our results suggest an important regulatory role of *PEMT* in vis AT and liver tissue, especially its expression in vis, as it correlated with the severity of steatosis and diabetes.

## Figures and Tables

**Table 1:** Association analyses of liver, visceral, and subcutaneous *PEMT* mRNA expression with anthropometric and metabolic parameters (n = 64).

	Sc AT *PEMT* mRNA *p*-Value adj (Beta; [95% CI])	Vis AT *PEMT* mRNA *p*-Value adj (Beta; [95% CI])	Liver *PEMT* mRNA *p*-Value adj (Beta; [95% CI])
Age (years)	0.5 (0.69; [−1.38 2.77])	0.59 (2.48; [−6.85 11.80])	0.5 (−0.33; [−1.29 0.64])
Body weight (kg)	0.09 (2.75; [−0.40 5.90])	0.75 (2.2; [−11.96 16.37])	0.58 (−0.44; [−2.03 1.16])
Height (m)	0.21 (0.01; [−0.01 0.03])	0.92 (0; [−0.07 0.06])	0.86 (0; [−0.007 0.008])
BMI (kg/m²)	0.26 (0.56; [−0.43 1.55])	0.57 (1.26; [−32,230 5.76])	0.35 (−0.23; [−0.71 0.25])
Waist circumference (cm)	**0.01 (2.28; [0.58 3.97])**	0.32 (3.89; [−3.95 11.72])	0.25 (−0.5; [−1.35 0.36])
Hip circumference (cm)	0.13 (1.25; [−0.40 2.89])	0.66 (−1.61; [−9.10 5.89])	0.74 (−0.14; [−0.95 0.68])
WHR	0.23 (0.01; [−0.01 0.03])	0.24 (0.04; [−0.03 0.11])	0.54 (0; [−0.01 0.01])
FPG (mmol/L)	0.32 (−0.35; [−1.04 0.35])	0.74 (−0.49; [−3.51 2.52])	0.55 (−0.08; [−0.33 0.18])
FPI (pmol/L)	0.93 (−1.11; [−26.53 24.31])	0.21 (−66.93; [−174.97 41.12])	0.3 (−3.57; [−10.40 3.25])
HbA1c (%)	0.3 (−0.23; [−0.67 0.21])	0.48 (−0.6; [−2.34 1.14])	0.7 (−0.03; [−0.19 0.13])
Total Cholesterol (mmol/L)	0.27 (−0.16; [−0.43 0.12])	0.62 (−0.27; [−1.37 0.84])	0.4 (−0.04; [−0.14 0.06])
HDL-C (mmol/L)	0.22 (−0.06; [−0.15 0.04])	0.93 (−0.02; [−0.39 0.35])	0.75 (0.01; [−0.03 0.04])
LDL-C (mmol/L)	0.38 (−0.1; [−0.34 0.14])	0.68 (−0.19; [−1.15 0.77])	0.25 (−0.05; [−0.14 0.04])
Triglycerides (mmol/L)	0.88 (−0.02; [−0.22 0.19])	0.13 (−0.58; [−1.36 0.20])	0.8 (−0.01; [−0.07 0.05])
CrP (mg/L)	0.74 (−0.26; [−1.77 1.26])	0.51 (2.23; [−4.65 9.11])	0.97 (0.01; [−0.70 0.73])
Bilirubin (µg/mL)	0.25 (0.79; [−0.56 2.14])	0.68 (1.24; [−4.98 7.47])	0.38 (−0.26; [−0.84 0.32])
gammaGT (µkat/L)	0.05 (0.16; [−0.002 0.33])	0.95 (−0.02; [−0.80 0.76])	0.2 (0.05; [−0.03 0.12])
AP (µkat/L)	0.45 (0.03; [−0.05 0.11])	0.73 (0.06; [−0.29 0.41])	0.07 (0.03; [−0.003 0.06])
ASAT (µkat/L)	0.33 (0.04; [−0.04 0.11])	0.44 (0.13; [−0.21 0.48])	0.83 (0; [−0.03 0.04])
ALAT (µkat/L)	0.7 (0.03; [−0.11 0.16])	0.93 (0.03; [−0.55 0.61])	0.54 (0.02; [−0.04 0.07])
Steatosis (%)	0.23 (0.02; [−0.02 0.06])	0.15 (0.13; [−0.05 0.31])	0.9 (0; [−0.01 0.01])
Liver Fat Content (mean)	0.19 (1.54; [−0.80 3.88])	**0.003 (14.53; [5.06 23.99])**	0.86 (−0.16; [−1.97 1.65])
Presence of:	*p*-value adj (OR; [95% CI])	*p*-value adj (OR; [95% CI])	*p*-value adj (OR; [95% CI])
NAFLD	N/A	N/A	N/A
NASH	0.22 (1.34; [0.84 2.16])	**0.01 (31.28; [1.87 522.09])**	0.32 (0.86; [0.64 1.16])
IGT	**0.03 (2.17; [1.10 4.31])**	0.16 (5.97; [0.48 74.85])	0.35 (0.86; [0.63 1.18])
Diabetes	**0.01 (2.16; [1.19 3.91])**	0.07 (8.08; [0.84 77.46])	0.4 (0.88; [0.66 1.18])
Hypertension	0.06 (3.35; [0.97 11.61])	0.08 (312.17; [0.46 212,038.25])	0.99 (1; [0.80 1.25])
Dyslipidemia	0.5 (1.17; [0.74 1.84])	0.16 (4.49; [0.55 36.83])	0.93 (1.01; [0.79 1.30])

*p*-values adj were calculated in linear regression or logistic regression after adjusting for age, sex and BMI. except for weight and BMI which was adjusted only for age and sex. Significant correlations (*p* < 0.05) are highlighted in bold. beta, effect size; OR, odd ratio; 95%[CI], 95% confidence interval; vis AT, visceral adipose tissue; sc AT, subcutaneous adipose tissue, liver tissue, BMI, body max index; FPG, fasting plasma glucose; FPI, fasting plasma insulin; HbA1c, hemoglobin A1c; HDL-C, high-density lipoprotein cholesterol; LDL-C, low-density lipoprotein cholesterol; SAT, subcutaneous adipose tissue; WHR, waist to hip; gamma GT, Gamma-glutamyltransferase; AP, alkaline phosphatase; ASAT, aspartate aminotransferase; ALAT, alanine aminotransferase; CRP, C-reactive protein; NAFLD, non-alcoholic fatty liver disease; NASH, non-alcoholic steatohepatitis; IGT, subjects with impaired glucose tolerance;. N/A, not applicable.

**Table 2:** Association of rs4646404 with *PEMT* mRNA expression in visceral (vis) and subcutaneous (sc) adipose tissue grouped by sex.

		rs4646404 G > A			*p*			p_adj_		
		GG	GA	AA	add	dom	rec	add	dom	rec
Total	ln*PEMT* sc	−0.6 (1.3)	−0.8 (1.4)	−0.9 (1.3)	0.07	0.33	0.07	0.15	0.65	0.1
	ln*PEMT* vis	−1.3 (1.6)	−1.7 (1.6)	−1.4 (1.9)	0.13	0.83	**0.03**	0.16	0.9	0.06
male	ln*PEMT* sc	−0.5 (1.3)	−0.8 (1.5)	−0.7 (0.9)	0.35	0.9	0.26	0.24	0.62	0.2
	ln*PEMT* vis	−1.2 (1.6)	−1.4 (1.3)	−1.3 (1.5)	0.73	0.96	0.69	0.19	0.85	0.49
female	ln*PEMT* sc	−0.6 (1.2)	−0.8 (1.3)	−1.0 (1.3)	0.12	0.3	0.15	0.34	0.85	0.27
	ln*PEMT* vis	−1.3 (1.6)	−1.8 (1.6)	−1.4 (1.9)	0.13	0.76	**0.03**	0.55	0.9	0.06

Data are given as arithmetic mean ± SEM. ln, logarithmic conversion; vis, visceral adipose tissue; sc, subcutaneous adipose tissue; add, additive model; dom, dominant model; rec, recessive model. P_adj_ for total means after adjusted BMI, sex, and age. p_adj_ for female and male means after adjusted BMI and age. Significant *p*-value is indicated in bold.

**Table 3:** Anthropometric and metabolic characteristics of the studied cohort.

	BMI < 30 kg/m^2^	BMI 30–40 kg/m^2^	BMI > 40 kg/m^2^
	(N = 242)	(N = 383)	(N = 2301)
Age (years)	61 ± 15	49 ± 12	46 ± 11
Female/Male (N)	127/115	273/110	1632/669
IGT/T2D (N)	39	139	1026
Body weight (kg)	73 ± 11.2	104.4 ± 14.7	145.9 ± 26.1
Height (m)	1.7 ± 0.1	1.7 ± 0.1	1.7 ± 0.1
BMI (kg/m^2^)	25.3 ± 2.5	36.3 ± 2.8	50.3 ± 7.2
Waist circumference (cm)	90.9 ± 17.4	116.6 ± 14.8	142.4 ± 17.4
Hip circumference (cm)	95.3 ± 11.8	118.3 ± 12.9	148.6 ± 16.7
WHR	0.9 ± 0.1	1 ± 0.1	1 ± 0.1
Body fat (%)	23.9 ± 5.1	38 ± 8.8	48 ± 8.4
FPG (mmol/L)	5.7 ± 1.2	6.2 ± 2.6	6.6 ± 2.7
FPI (pmol/L)	56.7 ± 75.5	109.3 ± 143	151.8 ± 136.2
HbA1c (%)	5.6 ± 0.7	5.9 ± 0.9	6.2 ± 1.3
Total cholesterol (mmol/L)	5.2 ± 1	5.1 ± 1.2	4.9 ± 1.1
HDL-C (mmol/L)	1.4 ± 0.4	1.3 ± 0.4	1.2 ± 0.5
LDL-C (mmol/L)	3.1 ± 0.9	3.3 ± 1	3.1 ± 0.9
Triglycerides (mmol/L)	1.2 ± 0.6	1.7 ± 1.1	1.9 ± 1.4
ALAT (µkat/L)	0.5 ± 0.6	0.8 ± 2.8	0.8 ± 2.5
ASAT (µkat/L)	0.4 ± 0.2	0.6 ± 0.3	0.6 ± 0.3
gamma-GT (µkat/L)	1.2 ± 1.9	0.9 ± 1.8	1 ± 6.8
Leptin (ng/mL)	10.9 ± 10.7	31.6 ± 17.8	59.1 ± 34.4
Sc/vis AT *PEMT* mRNA expression n (%)	21 (4)	48 (8)	505 (88)
rs4646404 (G > A) carrier n (%)	121 (10)	137 (11)	1011 (79)

Data are given as mean ± SD. AT: adipose tissue; IGT: impaired glucose tolerance; T2D: type 2 diabetes mellitus; BMI: body mass index; WHR: waist-to-hip-ratio; FPG: fasting plasma glucose; FPI: fasting plasma insulin; HbA1c: glycated hemoglobin; HDL-C: high-density lipoprotein cholesterol; LDL-C: low-density lipoprotein cholesterol; ALAT: alanine aminotransferase; ASAT: aspartate aminotransferase; n = number.

**Table 4:** Anthropometric and metabolic characteristics of the studied cohort.

	NAFLD	NASH	*p*-Value
	(N = 29)	(N = 35)	
Age (years)	45.45 ± 10.13	47.26 ± 10.06	0.239
Female/Male (N)	24/5	21/14	
IGT/T2D (N)	4/8	4/22	
Body weight (kg)	130.28 ± 18.76	136.05 ± 18.82	0.222
Height (m)	1.7 ± 0.11	1.7 ± 0.09	0.914
BMI (kg/m^2^)	44.93 ± 5.06	46.97 ± 4.65	0.095
Waist circumference (cm)	125.08 ± 13.26	136.90 ± 12.37	**<0.001**
Hip circumference (cm)	142.02 ± 11.8	140.25 ± 11.55	0.536
WHR	0.88 ± 0.08	0.98 ± 0.09	**<0.001**
Vis AT Volume (cm^3^)	5152.00 ± 2273.73	7211.31 ± 2951.50	**0.001**
Sc AT Volume (cm^3^)	19,265.75 ± 3565.77	18,600.33 ± 2605.83	0.707
Liver Volume (cm^3^)	1949.47 ± 372.70	2544.66 ± 540.29	**<0.001**
FPG (mmol/L)	5.19 ± 0.85	6.47 ± 2.29	**0.010**
FPI (pmol/L)	102.79 ± 74.70	119.13 ± 68.10	0.438
HbA1c (%)	5.21 ± 0.40	6.35 ± 1.51	**0.001**
Total cholesterol (mmol/L)	4.86 ± 0.97	4.35 ± 0.89	0.077
HDL-C (mmol/L)	1.25 ± 0.33	1.15 ± 0.25	0.268
LDL-C (mmol/L)	2.93 ± 0.86	2.51 ± 0.82	0.095
Triglycerides (mmol/L)	1.4 ± 0.64	1.48 ± 0.80	0.750
gammaGT (µkat/L)	0.65 ± 0.84	0.75 ± 0.55	0.594
AP (µkat/L)	1.32 ± 0.37	1.20 ± 0.30	0.174
ASAT (µkat/L)	0.59 ± 0.28	0.76 ± 0.34	**0.041**
ALAT (µkat/L)	0.78 ± 0.48	1.07 ± 0.58	**0.046**
Bilirubin (µg/mL)	9.08 ± 6.22	9.85 ± 5.48	0.610
CRP (mg/L)	8.26 ± 7.05	8.40 ± 7.87	0.943

Data are given as mean ± SD. IGT: impaired glucose tolerance; T2D: type 2 diabetes mellitus; BMI: body mass index; WHR: waist-to-hip-ratio; FPG: fasting plasma glucose; FPI: fasting plasma insulin; HbA1c: glycated hemoglobin; HDL-C: high-density lipoprotein cholesterol; LDL-C: low-density lipoprotein cholesterol; gammaGT: Gamma-glutamyltransferase; AP: alkaline phosphatase; ASAT: aspartate aminotransferase; ALAT: alanine aminotransferase; CRP: C-reactive protein; Significant *p*-values are indicated in bold.

## Supplementary Materials

The following supporting information can be downloaded at: https://www.mdpi.com/article/10.3390/ijms242316850/s1.
